# Association of depression symptom severity with short-term risk of an initial hospital encounter in adults with major depressive disorder

**DOI:** 10.1186/s12888-021-03258-3

**Published:** 2021-05-17

**Authors:** Jennifer Voelker, Kun Wang, Wenze Tang, Jinghua He, Ella Daly, Christopher D. Pericone, John J. Sheehan

**Affiliations:** 1 Janssen Scientific Affairs, LLC, 1125 Trenton Harbourton Rd, Titusville, NJ 08560 USA; 2 Janssen Research & Development, LLC, 1125 Trenton Harbourton Rd, Titusville, NJ 08560 USA

**Keywords:** Major depressive disorder, Depression symptom severity, PHQ-9 assessment, Healthcare resource utilization, Hospital encounter, Natural language processing

## Abstract

**Background:**

Despite the availability of pharmacologic and nonpharmacologic treatment options, depression continues to be one of the leading causes of disability worldwide. This study evaluated whether depression symptom severity, as measured by PHQ-9 score, of patients diagnosed with MDD is associated with short-term risk of a hospital encounter (ER visit or inpatient stay).

**Methods:**

Adults with ≥1 PHQ-9 assessment in an outpatient setting (index date) and ≥ 1 MDD diagnosis within 6 months prior were included from the de-identified Optum Electronic Health Record database (April 2016–June 2019). Patients were categorized by depression symptom severity based on PHQ-9 scores obtained by natural language processing. Crude rates, adjusted absolute risks, and adjusted relative risks of all-cause and MDD-related hospital encounters within 30 days following assessment of depression severity were determined.

**Results:**

The study population consisted of 280,145 patients with MDD and ≥ 1 PHQ-9 assessment in an outpatient setting. Based on PHQ-9 scores, 26.9% of patients were categorized as having none/minimal depression symptom severity, 16.4% as mild, 24.7% as moderate, 19.6% as moderately severe, and 12.5% as severe. Among patients with none/minimal, mild, moderate, moderately severe, and severe depression, the adjusted absolute short-term risks of an initial all-cause hospital encounter were 4.1, 4.4, 4.8, 5.6, and 6.5%, respectively; MDD-related hospital encounter adjusted absolute risks were 0.8, 1.0, 1.3, 1.6, and 2.1%, respectively. Compared to patients with none/minimal depression symptom severity, the adjusted relative risks of an all-cause hospital encounter were 1.60 (95% CI 1.50–1.70) for those with severe, 1.36 (1.29–1.44) for those with moderately severe, 1.18 (1.12–1.25) for those with moderate, and 1.07 (1.00–1.13) for those with mild depression symptom severity.

**Conclusions:**

These study findings indicate that depression symptom severity is a key driver of short-term risk of hospital encounters, emphasizing the need for timely interventions that can ameliorate depression symptom severity.

**Supplementary Information:**

The online version contains supplementary material available at 10.1186/s12888-021-03258-3.

## Background

Major depressive disorder (MDD) is a common and frequently recurrent psychiatric condition characterized by significant social and functional impairment [1, 2]. In 2018, 7.2% of adults in the United States reported they had experienced at least one major depressive episode in the prior year, with 65% reporting severe impairment [3]. In addition to an associated increased risk with poor health outcomes, including early onset and increased severity of chronic comorbid diseases and hospitalization [4–6], MDD is associated with an increased risk of suicidal ideation and suicide attempt [7, 8], as well as all-cause mortality [6].

The American Psychiatric Association has established 9 symptom criteria for diagnosis of MDD in the Diagnostic and Statistical Manual of Mental Disorders, Fifth Edition (DSM-5) [9]; the number, frequency, and intensity of symptoms can range from none/minimal to severe [9, 10]. Clinicians may use validated instruments as screening tools to assist in making the diagnosis of MDD, to quantify symptom severity, and to monitor ongoing symptom severity. In 2016, the US Preventive Services Task Force (USPSTF) recommended routine screening for depression among adults in the US and suggested the 9-item Patient Health Questionnaire (PHQ-9) for this purpose [11].

The most recent data available, which was collected prior to 2016 USPSTF routine depression screening recommendations, shows that screening for depression in the primary care setting in the US has in the past been widely underused [12, 13]. Based on National Ambulatory Care Surveys (NACS), Samples et al. reported only a 3.0% rate during years 2005 to 2015 of depression screening among visits to outpatient physician offices in the US [12]; the rate was reported at 4.2% in another study of physician-patient encounters based on 2012–2013 NACS data [13]. Patients with acute worsening of depression symptoms may present to the emergency room (ER), and the underuse of outpatient depression screening may contribute to this presentation. A study based on data from the Nationwide Emergency Department Sample reported that in 2014, ER visits for depression had increased by nearly 26% since 2006 in the US; over half of such ER visits in 2014 led to an inpatient stay that lasted on average 5.6 days [5]. In years 2014–2015 in the US, the average inpatient stay for MDD was 6 days with an average cost of $6713 [14]. The growing burden of mental illness on the healthcare system in the US is detailed in the 2017 report of the National Council for Behavioral Health Medical Director Institute, which notes that across all care settings (inpatient, outpatient, community, workforce, etc) there is insufficient access for those who are mentally ill [15]. In particular, there is an extensive and widespread psychiatric inpatient bed shortage, which contributes to exorbitantly long wait times in the ER for psychiatric care (up to 23 h on average for some dispositions) [15].

A better understanding of the impact of depression symptom severity on future use of healthcare resources, specifically that provided by hospitals, may be helpful to better determine the most appropriate targeted interventions that can be implemented earlier in the course of worsening depression symptoms. A few small-scale studies conducted in the US have found depression severity to impact use of healthcare resources, including hospital encounters and mental health services [16, 17]. Adekkanattu et al. recently developed a natural language processing (NLP) method to extract PHQ-9 scores from unstructured data in electronic health records (EHRs) that exhibited high accuracy (97%) and sensitivity (98%) compared to a reference standard to identify patients with MDD and had utility for stratifying patients by depression severity [18]. Utilizing a similar NLP approach to extract PHQ-9 assessment data developed by Optum (Eden Prairie, MN), in this study we evaluated whether depression symptom severity, as measured by PHQ-9 score, in a large US population of patients diagnosed with MDD is associated with short-term risk of a hospital encounter.

## Methods

### Data source and study population

Utilizing data from the Optum® EHR database, this study retrospectively analyzed healthcare resource utilization (HRU) outcomes of patients diagnosed with MDD who had a PHQ-9 assessment administered in an outpatient care setting. The Optum EHR database is a multidimensional database that contains information on outpatient visits, diagnostic procedures, medications, laboratory results, hospitalizations, clinical notes, and patient outcomes primarily from integrated delivery networks. The database includes data from > 80 million patients, with ≥7 million patients from each US census region that is captured by a network of ≥140,000 providers at > 700 hospitals and > 7000 clinics. Both structured and unstructured data from the Optum EHR database were used in the analyses of this study. The structured data included demographic information, clinical characteristics, and HRU. PHQ-9 scores were determined from unstructured data derived from EHR note fields using Optum’s proprietary NLP. All patient data contained in the Optum EHR database are de-identified and in compliance with the Health Insurance Portability and Accountability Act.

Adult patients (≥18 years of age) with ≥1 record of a PHQ-9 assessment in an outpatient care setting (index date) during April 2016 to June 2019 were included from the de-identified Optum EHR database. Individuals may have had multiple PHQ-9 assessments and only the first such assessment was selected with the corresponding date defined as the index date. Patients were required to have ≥1 diagnosis of MDD (International Classification of Diseases, 10th revision [ICD-10] codes provided in supplementary material) on or during the 6 months prior to the index date and ≥ 6 months of health activity in the Optum EHR database preceding the index date. The baseline period was defined as the 6 months prior to the index date; the follow-up period was defined as the 30 days following the index date. Patients were excluded if they had a diagnosis during any timepoint in the study period of bipolar disorder, schizophrenia or other non-mood related psychotic disorders, dementia, and intellectual disability.

### Assessment of depression severity

The PHQ-9, a patient self-reported instrument, is aligned with the DSM-5 symptom criteria for MDD and has been validated as a useful tool for the screening of depressive disorders and as a reliable and valid measure of depression symptom severity by a multitude of studies [19–21]. Patients were assigned to the following study cohorts based on their PHQ-9 score (range from 0 to 27) defined by Kroenke et al. [19]: None/minimal = score 0 to 4, mild = score 5 to 9, moderate = score 10 to 14, moderately severe = score 15 to 19, and severe = score 20 to 27. If multiple PHQ-9 assessments were recorded on the same day, the highest score was used to categorize patients into study cohorts.

PHQ-9 scores in the Optum EHR database are extracted from physician notes via NLP. We excluded original values that appeared invalid using criteria defined in collaboration with Optum. Optum further ascertained the accuracy of the remaining PHQ-9 scores in 100 random samples in comparison to manual curation. After matching by subject ID and date, 99 out of the 100 NLP-extracted PHQ-9 scores matched exactly to the manually curated PHQ-9 scores.

### Patient demographics and clinical characteristics

Patient demographics, including age, gender, race, ethnicity, US geographic region of residence, and insurance type, and clinical characteristics were evaluated for each patient eligible for the study during the baseline period or on the index date and are reported for the overall study population and stratified by depression symptom severity level study cohorts. The clinical characteristics evaluated included smoking status, past suicidal ideation, past suicide attempt, psychiatric comorbidities based on the DSM-5 [9], including, anxiety disorders, sleep-wake disorders, trauma-and-stressor-related disorders, caffeine and nicotine addictions, all other substances addiction (non-caffeine and tobacco) [22, 23], depression treatment, and Elixhauser comorbidity index score. The Elixhauser comorbidity index is a risk adjustment tool used to categorize 30 comorbidities based on ICD diagnosis codes available in administrative data and can be used to predict hospital resource use and in-house hospital mortality [24, 25]. Psychiatric comorbidities were also based on ICD diagnosis codes and depression treatment (antidepressants and psychotherapy) was based on Generic Product Identifiers, National Drug Codes, or Current Procedural Terminology Codes, all found within the structured data of the Optum EHR database.

### HRU outcome measurements

During the 30 days following patients’ PHQ-9 assessments (ie, in the short-term), the proportions of patients with initial all-cause and MDD-related hospital encounters were evaluated. MDD-related HRU was defined as a hospital encounter with at least one MDD ICD-10 code identified during the period of inpatient stay or ER visit. Given the limitations of identifying primary diagnoses in the data source, both primary and secondary diagnoses were used to define the event as MDD-related.

### Statistical analyses

Descriptive statistics were used to summarize demographics, clinical characteristics, and crude rates of HRU outcomes with means and standard deviations (SDs) for continuous variables and counts and percentages for categorical variables. A marginal structural model was created using a generalized linear model with binomial distribution and stabilized inverse probability weighting to estimate adjusted absolute risks at each depression symptom severity level under identity link and causal risk ratios of HRU outcomes (all-cause and MDD-related initial hospital encounters following a PHQ-9 assessment) across the depression symptom severity levels under log link (risk ratio [RR] with 95% confidence intervals [CI] were reported). Due to the observational nature of the study design and introduction of bias due to the lack of randomization, stabilized inverse probability weights were generated with multinomial logistic regression, adjusting for potential confounders associated with the risk of a hospital encounter, including baseline demographics (age, gender, race, ethnicity, US geographic region of residence, and insurance type) and clinical characteristics (smoking status, psychiatric comorbidities based on the DSM-5 [9], depression treatment, and nonpsychiatric individual Elixhauser comorbidities).

## Results

### Study population

The overall study population consisted of 280,145 patients with MDD and ≥ 1 PHQ-9 assessment in an outpatient care setting; 26.9% were categorized as having none/minimal depression symptom severity, 16.4% as mild, 24.7% as moderate, 19.6% as moderately severe, and 12.5% as severe. Demographics and selected clinical characteristics of the overall patient population and study cohorts stratified by depression symptom severity level are shown in Table 1. As depression symptom severity level increased, mean ages decreased across the study cohorts (none/minimal: 53.7 years; mild: 48.8 years; moderate: 47.1 years; moderately severe: 44.0 years; severe: 42.6 years, *p* < 0.001); correspondingly, the distribution of patients shifted to younger age groups. Across the study cohorts, approximately 72–73% of patients were female, 80–89% were Caucasian, 60–68% had residence in the Midwest, and 21–30% had commercial insurance coverage.

**Table 1:** Demographics and selected clinical characteristics of overall patient population and study cohorts

	*Overall Population*	*None/Minimal*	*Mild*	*Moderate*	*Moderately Severe*	*Severe*
	*N (%)*	*N (%)*	*N (%)*	*N (%)*	*N (%)*	*N (%)*
	*280,145 (100)*	*75,227 (26.9)*	*45,857 (16.4)*	*69,325 (24.7)*	*54,772 (19.6)*	*34,964 (12.5)*
*Age (years)*						
Mean (SD)	48.0 (17.6)	53.7 (17.2)	48.8 (17.5)	47.1 (17.6)	44.0 (16.9)	42.6 (15.9)
*Age distribution*						
18–25 years	36,211 (12.9)	5410 (7.2)	5192 (11.3)	9608 (13.9)	9653 (17.6)	6348 (18.2)
26–34 years	40,319 (14.4)	7357 (9.8)	6354 (13.9)	10,690 (15.4)	9480 (17.3)	6438 (18.4)
35–44 years	44,813 (16.0)	10,095 (13.4)	7605 (16.6)	11,296 (16.3)	9407 (17.2)	6410 (18.3)
45–54 years	49,505 (17.7)	12,890 (17.1)	8131 (17.7)	11,988 (17.3)	9795 (17.9)	6701 (19.2)
55–64 years	53,410 (19.1)	16,422 (21.8)	8986 (19.6)	12,590 (18.2)	9531 (17.4)	5881 (16.8)
65+ years	55,887 (19.9)	23,053 (30.6)	9589 (20.9)	13,153 (19.0)	6906 (12.6)	3186 (9.1)
*Gender*						
Female	202,990 (72.5)	54,572 (72.5)	33,256 (72.5)	49,886 (72.0)	39,766 (72.6)	25,510 (73.0)
Male	77,155 (27.5)	20,655 (27.5)	12,601 (27.5)	19,439 (28.0)	15,006 (27.4)	9454 (27.0)
*Race*						
Caucasian	238,847 (85.3)	66,866 (88.9)	39,905 (87.0)	58,925 (85.0)	45,316 (82.7)	27,835 (79.6)
African American	21,961 (7.8)	4789 (6.4)	3000 (6.5)	5270 (7.6)	5036 (9.2)	3866 (11.1)
Asian	2475 (0.9)	530 (0.7)	390 (0.9)	619 (0.9)	534 (1.0)	402 (1.1)
Other	16,862 (6.0)	3042 (4.0)	2562 (5.6)	4511 (6.5)	3886 (7.1)	2861 (8.2)
*Ethnicity*						
Non-Hispanic	245,525 (87.6)	68,592 (91.2)	40,764 (88.9)	59,055 (85.2)	47,260 (86.3)	29,854 (85.4)
Hispanic	12,915 (4.6)	2183 (2.9)	1991 (4.3)	3232 (4.7)	3312 (6.0)	2197 (6.3)
Unknown	21,705 (7.7)	4452 (5.9)	3102 (6.8)	7038 (10.2)	4200 (7.7)	2913 (8.3)
*US geographic region*						
Midwest	177,679 (63.4)	51,231 (68.1)	29,178 (63.6)	41,902 (60.4)	33,758 (61.6)	21,610 (61.8)
Northeast	20,950 (7.5)	6289 (8.4)	4162 (9.1)	4776 (6.9)	3582 (6.5)	2141 (6.1)
South	41,404 (14.8)	9618 (12.8)	6520 (14.2)	10,964 (15.8)	8985 (16.4)	5317 (15.2)
West	27,161 (9.7)	4953 (6.6)	4123 (9.0)	8583 (12.4)	5603 (10.2)	3899 (11.2)
Other/unknown	12,951 (4.6)	3136 (4.2)	1874 (4.1)	3100 (4.5)	2844 (5.2)	1997 (5.7)
*Insurance type*						
Commercial	72,587 (25.9)	16,111 (21.4)	10,039 (21.9)	20,285 (29.3)	16,244 (29.7)	9908 (28.3)
Medicaid	11,682 (4.2)	1467 (2.0)	1354 (3.0)	3242 (4.7)	3095 (5.7)	2524 (7.2)
Medicare	28,535 (10.2)	11,406 (15.2)	4162 (9.1)	6732 (9.7)	4050 (7.4)	2185 (6.2)
Multiple	107,215 (38.3)	32,404 (43.1)	19,914 (43.4)	24,474 (35.3)	18,387 (33.6)	12,036 (34.4)
Other payer types	4267 (1.5)	1049 (1.4)	669 (1.5)	1027 (1.5)	890 (1.6)	632 (1.8)
Uninsured	2592 (0.9)	302 (0.4)	278 (0.6)	695 (1.0)	746 (1.4)	571 (1.6)
Missing	18,271 (6.5)	3384 (4.5)	3094 (6.7)	4661 (6.7)	4520 (8.3)	2612 (7.5)
Unknown	34,996 (12.5)	9104 (12.1)	6347 (13.8)	8209 (11.8)	6840 (12.5)	4496 (12.9)
*Smoking status*						
Current smoker	77,927 (27.8)	16,745 (22.3)	11,424 (24.9)	19,764 (28.5)	17,299 (31.6)	12,695 (36.3)
Non-smoker	77,626 (27.7)	22,181 (29.5)	13,424 (29.3)	18,348 (26.5)	14,847 (27.1)	8826 (25.2)
History of smoking	124,592 (44.5)	36,301 (48.3)	21,009 (45.8)	31,213 (45.0)	22,626 (41.3)	13,443 (38.4)
*Past suicidal ideation*	4443 (1.6)	723 (1.0)	548 (1.2)	915 (1.3)	964 (1.8)	1293 (3.7)
*Past suicide attempt*	1451 (0.5)	265 (0.4)	229 (0.5)	326 (0.5)	327 (0.6)	304 (0.9)
*Elixhauser index score*						
Mean (SD)	13.4 (14.2)	14.6 (14.9)	13.4 (14.2)	13.2 (14.3)	12.5 (13.6)	12.4 (13.4)
*Psychiatric comorbidities*^a^						
Anxiety disorders	143,705 (51.3)	33,819 (45.0)	23,609 (51.5)	35,923 (51.8)	29,908 (54.6)	20,446 (58.5)
Sleep-wake disorders	43,787 (15.6)	12,651 (16.8)	7414 (16.2)	10,979 (15.8)	7893 (14.4)	4850 (13.9)
Caffeine and nicotine addictions	38,999 (13.9)	8086 (10.7)	5716 (12.5)	10,188 (14.7)	8790 (16.0)	6219 (17.8)
Trauma-and-stressor-related disorders	26,747 (9.5)	5547 (7.4)	4410 (9.6)	6534 (9.4)	5859 (10.7)	4397 (12.6)
All other substances addiction (non-caffeine and tobacco)	17,926 (6.4)	3796 (5.0)	2968 (6.5)	4336 (6.3)	3887 (7.1)	2939 (8.4)
*Depression treatment*						
Antidepressant	215,130 (76.8)	55,463 (73.7)	34,269 (74.7)	53,443 (77.1)	43,804 (80.0)	28,151 (80.5)
Psychotherapy	49,527 (17.7)	10,867 (14.4)	9526 (20.2)	11,769 (17.0)	10,166 (18.6)	7469 (21.4)

Across all listed comparisons of the study cohorts stratified by depression symptom severity level, *p*-values were < 0.001

^a^Reported for psychiatric comorbidities present in the overall study population at > 5%

*MDD* Major depressive disorder, *SD* Standard deviation

In study cohorts with greater depression symptom severity, the proportions of patients who were African American, Asian, and other races were higher than observed in study cohorts with lower depression symptom severity; similarly, the proportions of patients with Hispanic ethnicity were also higher in study cohorts with greater depression symptom severity. The proportions of patients with Medicaid coverage also were higher among study cohorts with moderate (4.7%), moderately severe (5.7%), and severe (7.2%) depression than among study cohorts with lower levels of depression symptom severity (2.0–3.0%).

More patients with moderate (suicidal ideation: 1.3%; suicide attempt: 0.5%), moderately severe (suicidal ideation: 1.8%; suicide attempt: 0.6%), and severe (suicidal ideation: 3.7%; suicide attempt: 0.9%) depression had documentation of past suicidal ideation and suicide attempt compared to study cohorts with lower levels of depression symptom severity. Among the overall patient population, the mean Elixhauser index score was 13.4; this mean score decreased as depression symptom severity level increased. The comorbid psychiatric disorder that was the most prevalent among the overall study population was anxiety disorder (51.3%), which increased in prevalence as depression symptom severity level increased.

### HRU outcome measurements

**Fig. 1:**
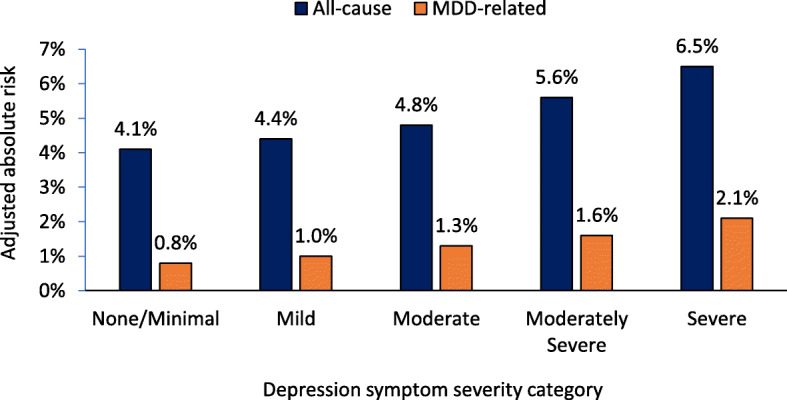
Adjusted absolute risks of all-cause and MDD-related initial hospital encounters following a PHQ-9 assessment. MDD: Major depressive disorder

The crude rates and adjusted absolute risks of all-cause and MDD-related initial hospital encounters in the short-term following a PHQ-9 assessment are shown in Table 2 and Fig. 1, respectively. Across all the evaluated types of initial hospital encounters in the short-term following a PHQ-9 assessment, crude rates increased with the level of depression symptom severity. When accounting for differences in baseline characteristics that may impact HRU, the adjusted results were consistent with the crude results. Among patients with none/minimal, mild, moderate, moderately severe, and severe depression, the adjusted absolute risks (95% CI) of an all-cause hospital encounter were 4.1% (3.9–4.2%), 4.4% (4.2–4.6%), 4.8% (4.7–5.0%), 5.6% (5.4–5.8%), and 6.5% (6.3–6.8%), respectively; of an MDD-related hospital encounter they were 0.8% (0.7–0.9%), 1.0% (0.9–1.1%), 1.3% (1.2–1.4%), 1.6% (1.5–1.6%), and 2.1% (2.0–2.3%), respectively.

**Table 2:** Crude rates of all-cause and MDD-related initial hospital encounters following a PHQ-9 assessment

	*None/Minimal*	*Mild*	*Moderate*	*Moderately severe*	*Severe*
*No. of patients*	75,227	45,857	69,325	54,772	34,964
*Type of hospital encounter, N (%)*					
All-cause	2746 (3.7)	1927 (4.2)	3363 (4.9)	3026 (5.5)	2369 (6.8)
MDD-related	591 (0.8)	452 (1.0)	921 (1.3)	813 (1.5)	776 (2.2)

*MDD* Major depressive disorder

**Fig. 2:**
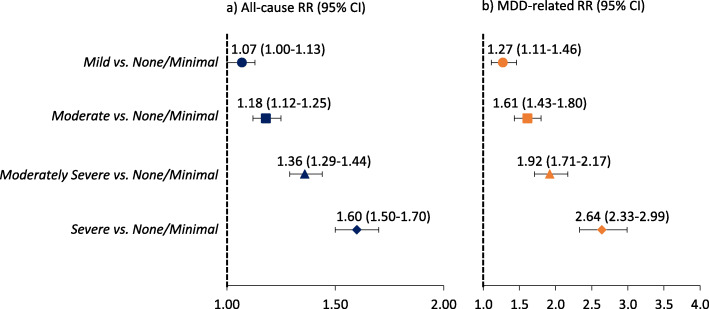
Adjusted relative risks of a) all-cause and b) MDD-related initial hospital encounters following a PHQ-9 assessment. *P*-values for all comparisons were < 0.05. For panels a and b, the x-axes are in different scales. The potential confounders adjusted for included baseline demographics (age, gender, race, ethnicity, US geographic region of residence, and insurance type) and clinical characteristics (smoking status, psychiatric comorbidities based on the DSM-5 [9], depression treatment, and nonpsychiatric individual Elixhauser comorbidities). CI: Confidence interval; MDD: Major depressive disorder; RR: Relative risk

The adjusted RRs of all-cause and MDD-related initial hospital encounters in the short-term following a PHQ-9 assessment are shown in Fig. 2 (each depression symptom severity level cohort compared to none/minimal) and supplementary Table 1 (all depression symptom severity level cohort comparisons). Compared to patients with none/minimal depression symptom severity, those with mild depression had a 7% increased risk of an all-cause initial hospital encounter in the short-term following their PHQ-9 assessment, those with moderate depression had an 18% increased risk, those with moderately severe depression had a 36% increased risk, and those with severe depression had a 60% increased risk (all comparisons were *p* < 0.05). This trend of a significantly increased risk of an initial hospital encounter associated with increased depression symptom severity level vs. none/minimal was consistent for MDD-related hospital encounters. Furthermore, the increased risk of an initial all-cause hospital encounter was apparent across all depression symptom severity level comparisons (ie, moderate vs. mild, moderately severe vs. mild, severe vs. mild, etc.) and consistent for MDD-related hospital encounters.

## Discussion

This is the first large-scale study in the US to evaluate the association between depression symptom severity and short-term risk of hospital encounters of patients with MDD. Among more than 280,000 US adult patients diagnosed with MDD who had a PHQ-9 assessment in an outpatient care setting, 26.9% were categorized as having none/minimal depression symptom severity, 16.4% as mild, 24.7% as moderate, 19.6% as moderately severe, and 12.5% as severe. A notable finding of this study was the nearly stepwise manner that depression symptom severity was associated with an increased risk of an MDD-related hospital encounter in the short-term following a PHQ-9 assessment; the increased risk ranged from 27 to 164% among those with mild to severe depression symptom severity compared to those with none/minimal. These study findings were obtained after adjustment for differences in patient demographics and clinical characteristics, including presence of comorbid psychiatric disorders and receipt of depression treatment, indicating that depression symptom severity is a key driver of the short-term risk of presentation to the hospital setting. Moreover, our study findings imply that taking steps towards improving depression symptoms, even if by only one severity category, may be helpful to incrementally reduce the risk of short-term HRU, particularly among those in the highest severity categories.

The findings of this study significantly contribute to the accumulating evidence of the association of depression symptom severity with increased HRU in the US observed in a few other studies conducted on a considerably smaller scale of patients with MDD [16, 17]. Beiser et al. conducted a prospective cohort study of 999 adults who presented to the ER for care other than for a psychiatric illness at a single US academic medical center in 2015 [16]. Based on depression diagnostic screening and depression severity measurement administered via a tablet computer during the ER visit, patients identified with MDD (27%) had a significantly greater risk of a subsequent ER visit (61% increased risk) and hospitalization (49% increased risk) during the 1 year follow-up period than those without MDD; furthermore, each 10% increase in MDD severity was associated with a 10% greater relative risk of a subsequent hospital encounter [16]. In another study of 539 individuals who self-rated their MDD using the Quick Inventory of Depressive Symptomatology Self-Report (2001–2002), 13.8% were classified as having mild depression, 38.5% as moderate, and 47.7% as severe; those with moderate and severe MDD used mental health services to a greater extent than those with mild disease, as well as had greater prevalence of unemployment, reduced work productivity, and disability [17]. A relatively larger study of 10,443 individuals in Stockholm, Sweden (1998–2014) reported that those with subsyndromal, mild, moderate, and severe depression, according to scores on the Major Depression Inventory, had higher incidence rates of mental health-related hospitalizations and outpatient care visits than non-depressed individuals; similar to our study, Sun et al. reported that as depression severity level increased, so did the rate of HRU [26].

Another remarkable finding of this study pertains to the association of increased depression symptom severity with increased risk of all-cause hospital encounters. Compared to patients with MDD and none to minimal depression symptom severity, according to index PHQ-9 assessments, those with mild symptoms had a 7% increased risk of an all-cause hospital encounter in the short-term following their PHQ-9 assessment, those with moderate had an 18% increased risk, those with moderately severe had a 36% increased risk, and those with severe had a 60% increased risk. This stepwise association of depression symptom severity level with increased relative risk of an all-cause hospital encounter in the short-term following a PHQ-9 assessment was observed after adjustment for age and the presence of Elixhauser comorbidities. Reviewed in Kessler et al. [6], a number of other studies have previously shown that MDD is associated with early onset and/or increased severity of several comorbid chronic illnesses, including cardiovascular disease, diabetes, respiratory illnesses, and chronic pain. Reported in a systematic review of several studies, depressive symptoms are a significant predictor of general hospital admissions for non-psychiatric reasons (RR: 1.36, 95% CI: 1.28–1.44), as well as longer inpatient lengths of stay and higher readmission risk [27]. The findings of our study add to this existing evidence of the association of MDD with increased HRU for non-psychiatric reasons by demonstrating the impact of increased depression symptom severity on the increased likelihood of all-cause HRU in the short-term. The reasons surrounding the association of depression symptom severity with greater utilization of healthcare resources in general are not well understood, but could be related to suboptimal treatment options, inadequate adherence to medications or treatments, a greater prevalence of chronic conditions (eg, substance abuse disorders, smoking history), lack of follow-up care by patients and/or providers, sociodemographic disparities, access to psychiatric care facilities, etc.

Two other studies conducted in the last decade in the US have also examined the distribution of patients with MDD across symptom severity categories based on PHQ-9 assessments, although they did not explore HRU to a significant extent [28, 29]. In a study of 1019 patients diagnosed with MDD (2006–2010 population sample from 9 US states in different geographic regions), Valuck found a generally similar distribution of patients with MDD across PHQ-9 score categories [28]. In another population of patients with MDD in the US (*N* = 315, 2014–2016), Bushnell et al. reported 6.7% of patients with none/minimal (PHQ-9 score: 0–4), 26.7% with mild (PHQ-9 score: 5–9), 21.9% with moderate (PHQ-9 score: 10–14), 27.6% with moderately severe (PHQ-9 score: 15–19), and 17.1% with severe (PHQ-9 score: 20–27) depression [29]. In our study, PHQ-9 scores of patients diagnosed with MDD were documented in EHRs, and therefore likely do not fully represent the distribution of severity categories among the overall population of patients with MDD in the US, especially those who have not been diagnosed and/or are without access to routine healthcare. Furthermore, a large proportion of patients represented in this study were from the Midwest region of the US; this particular region also contributed disproportionately to the none/mild depression symptom severity category. These findings may suggest that the PHQ-9 is used more routinely and that there is greater access to depression treatment in the Midwest compared to other US regions. Additionally, African American patients with MDD disproportionately were in the highest depression symptom severity categories. Several factors could contribute to this finding, including reduced access to routine healthcare resources where lower severity MDD may be assessed and documented. Nevertheless, the distribution of MDD patients across the PHQ-9 categories observed in this analysis broadly matches that observed in other studies with much smaller populations [28, 29].

This study has strengths in that it included a very large population of patients diagnosed with MDD across the US who had a PHQ-9 assessment, a validated instrument for measuring depression symptom severity [19]. Since the PHQ-9 has been shown to have good agreement with clinician depression rating scales (eg, Hamilton Depression Rating Scale) commonly used in clinical trial settings [30], the population-level data herein may be useful to apply to clinical trial data for economic modeling and other treatment-related measurement purposes. Furthermore, Optum’s NLP technology allowed for the timely extraction of large amount of PHQ-9 score data from complex clinical notes available in the EHRs. In addition, the marginal structural model (MSM) allowed us to identify the adjusted absolute risk at each level of severity level, which is not possible with conventional regression techniques. MSM is also agnostic about the effect modification by any covariates, therefore the relative risk estimates are not subject to model misspecification with respect to any product terms between depression severity and baseline covariates that could be possibly mis-specified in conventional regression techniques.

### Limitations of the study

The findings of this study should be interpreted with the understanding of certain limitations. First, the Optum EHR database may not contain all patient diagnoses and interactions with the healthcare system, including visits for psychiatric care provided by specialists who do not contribute to EHRs within the database. This study only included individuals with a recorded MDD diagnosis captured via an ICD code and also a PHQ-9 score available; thus, results may not generalize to individuals beyond this population, such as those with a high PHQ-9 score but no recorded diagnosis of MDD. Other potential limitations of the Optum EHR database include the inability distinguish primary versus secondary reasons for hospitalization, and thus we cannot assert that MDD was the primary reason for MDD-related HRU, but can conclude that the encounter was related to MDD as the diagnosis was recorded at some point during the hospital encounter. Second, since depression severity varies over time, using a single time point measure as a proxy of a complex exposure trajectory may result in non-differential measurement error that biases our results towards the null. As an observational study, no causal relationship between depression symptom severity and short-term HRU can be affirmed; however, such a hypothesis is plausible and can guide actions since a randomization of similar populations is not possible. Also, since the findings were based on real-world data obtained from EHRs, there is the potential for inaccuracies in diagnosis codes, missing records, and erroneous PHQ-9 scores recorded by healthcare providers in the clinical notes. However, our validation process confirmed that the NLP technology generated accurate PHQ-9 score outputs. Further study using similar methodology across other EHR database sources is warranted.

## Conclusions

This study of over 280,000 adults with MDD in the US is the largest study to date to evaluate how depression symptom severity, according to PHQ-9 assessments during an outpatient appointment, impacts the likelihood of presenting to a hospital in the short-term. The study findings highlight the nearly stepwise manner that depression symptom severity is associated with increased risk of initial all-cause and MDD-related hospital encounters in the 30 days following an outpatient PHQ-9 assessment. Additionally, they emphasize the importance of routine depression screening and timely intervention with efficacious treatments, including pharmacotherapy, psychotherapy, and other types of individualized patient management strategies potentially implemented in the outpatient setting, as prevention tactics to mitigate the course of acute worsening of symptoms of MDD and reduce some of the burden currently incurred by hospital systems.

## Supplementary Information

**Additional file 1: Supplementary Table 1.** Adjusted relative risks of initial hospital encounters in the short-term following a PHQ-9 assessment. **Supplementary Table 2.** ICD-10 codes and descriptions for MDD diagnoses.

## Data Availability

All data for this study are contained within the article and online supplementary material; Elixhauser nonpsychiatric individual comorbidity data are available from the corresponding author on reasonable request.

## Abbreviations

CI: Confidence interval
DSM-5: Diagnostic and Statistical Manual of Mental Disorders, Fifth Edition
EHR: Electronic health record
ER: Emergency room
HRU: Healthcare resource utilization
ICD-10: International Classification of Diseases, 10th revision
MDD: Major depressive disorder
MSM: Marginal structural model
NACS: National Ambulatory Care Survey
NLP: Natural language processing
PHQ-9: 9-item Patient Health Questionnaire
RR: Risk ratio
SD: Standard deviation
USPSTF: US Preventive Services Task Force
