# Supplementary material for: Association of depression symptom severity with short-term risk of an initial hospital encounter in adults with major depressive disorder
Source: BMC Psychiatry. 2021 May 17;21:257. doi: 10.1186/s12888-021-03258-3 (PMC8130130; doi:10.1186/s12888-021-03258-3)
Supplement: Supplementary file 1 — Additional file 1: Supplementary Table 1. Adjusted relative risks of initial hospital encounters in the short-term following a PHQ-9 assessment. Supplementary Table 2. ICD-10 codes and descriptions for MDD diagnoses. [file 12888_2021_3258_MOESM1_ESM.docx]

**Supplementary material**

**Supplementary Table 1** Adjusted relative risks of initial hospital encounters in the short-term following a PHQ-9 assessment

| *Type of hospital encounter* | *Comparison* | *RR* | *Lower*  *95% CI* | *Upper 95% CI* |
| --- | --- | --- | --- | --- |
| *All-cause* | |  |  |  |
|  | Mild vs. None/Minimal | 1.07 | 1.00 | 1.13 |
|  | Moderate vs. None/Minimal | 1.18 | 1.12 | 1.25 |
|  | Moderately Severe vs. None/Minimal | 1.36 | 1.29 | 1.44 |
|  | Severe vs. None/Minimal | 1.60 | 1.50 | 1.70 |
|  | Moderate vs. Mild | 1.11 | 1.05 | 1.17 |
|  | Moderately Severe vs. Mild | 1.28 | 1.21 | 1.35 |
|  | Severe vs. Mild | 1.50 | 1.41 | 1.60 |
|  | Moderately Severe vs. Moderate | 1.16 | 1.10 | 1.21 |
|  | Severe vs. Moderate | 1.35 | 1.28 | 1.43 |
|  | Severe vs. Moderately Severe | 1.17 | 1.11 | 1.24 |
| *MDD-related* | |  |  |  |
|  | Mild vs. None/Minimal | 1.27 | 1.11 | 1.46 |
|  | Moderate vs. None/Minimal | 1.61 | 1.43 | 1.80 |
|  | Moderately Severe vs. None/Minimal | 1.92 | 1.71 | 2.17 |
|  | Severe vs. None/Minimal | 2.64 | 2.33 | 2.99 |
|  | Moderate vs. Mild | 1.26 | 1.13 | 1.42 |
|  | Moderately Severe vs. Mild | 1.51 | 1.34 | 1.70 |
|  | Severe vs. Mild | 2.08 | 1.84 | 2.35 |
|  | Moderately Severe vs. Moderate | 1.20 | 1.09 | 1.32 |
|  | Severe vs. Moderate | 1.64 | 1.48 | 1.82 |
|  | Severe vs. Moderately Severe | 1.37 | 1.23 | 1.53 |

CI: Confidence interval; MDD: Major depressive disorder; RR: Relative risk

**Supplementary Table 2** ICD-10 codes and descriptions for MDD diagnoses

| *Code* | *Description* |
| --- | --- |
| F32 | Major depressive disorder, single episode |
| F32.0 | Major depressive disorder, single episode, mild |
| F32.1 | Major depressive disorder, single episode, moderate |
| F32.2 | Major depressive disorder, single episode, severe without psychotic features |
| F32.3 | Major depressive disorder, single episode, severe with psychotic features |
| F32.4 | Major depressive disorder, single episode, in partial remission |
| F32.5 | Major depressive disorder, single episode, in full remission |
| F32.9 | Major depressive disorder, single episode, unspecified |
| F33 | Major depressive disorder, recurrent |
| F33.0 | Major depressive disorder, recurrent, mild |
| F33.1 | Major depressive disorder, recurrent, moderate |
| F33.2 | Major depressive disorder, recurrent severe without psychotic features |
| F33.3 | Major depressive disorder, recurrent, severe with psychotic symptoms |
| F33.4 | Major depressive disorder, recurrent, in remission |
| F33.40 | Major depressive disorder, recurrent, in remission, unspecified |
| F33.41 | Major depressive disorder, recurrent, in partial remission |
| F33.42 | Major depressive disorder, recurrent, in full remission |
| F33.9 | Major depressive disorder, recurrent, unspecified |
